# O valor simbólico do uso de droga para população em situação de rua: droga que mata e alimenta

**DOI:** 10.1590/0102-311XPT173323

**Published:** 2024-11-04

**Authors:** Claudia Brito, Valeska Holst Antunes

**Affiliations:** 1 Escola Nacional de Saúde Pública Sergio Arouca, Fundação Oswaldo Cruz, Rio de Janeiro, Brasil.; 2 Faculdade de Medicina, Universidade Federal do Rio de Janeiro, Rio de Janeiro, Brasil.

**Keywords:** Drogas Ilícitas, Pessoas Mal Alojadas, Redução do Dano, Serviços de Saúde, Fome, Illicit Drugs, Ill-Housed Persons, Harm Reduction, Health Services, Hunger, Drogas Ilícitas, Pessoas Mal Alojadas, Reducción del Daño, Servicios de Salud, Hambre

## Abstract

Desde a antiguidade, consome-se drogas e seu tabu promoveu segregações e inibiu o enfrentamento adequado do uso nocivo. Este trabalho objetivou descortinar valores reais e simbólicos do uso de drogas pela população em situação de rua para além da patologização, buscando entender fatores que dificultam sua renúncia, mesmo com perdas e sofrimentos intensos. Este estudo visou também compreender as implicações do uso para o cuidado de saúde. Esta é uma pesquisa qualitativa fenomenológica, envolvendo observação participante do atendimento da população em situação de rua pelo Consultório na Rua, entrevistas com população em situação de rua e grupo focal com profissionais da Rede de Atenção à Saúde, em território do Município do Rio de Janeiro, Brasil, entre março de 2017 a julho de 2019. Resultados mostraram que “uso de droga” teve interação com as categorias da pesquisa da seguinte forma: droga é o principal motivo para viver e manter-se na rua; motiva a ter atividade financeira; faz abandonar tratamento de saúde; aplaca e provoca depressão, sofrimento e fome; faz buscar abrigo e religião, para “fugir” das drogas mas sem o tratamento adequado, esperança transverte-se em recaída e baixa autoestima, da qual deseja livrar-se, no entanto, poucos êxitos minam a esperança. Correcionalmente, é motivo das divergências entre profissionais quanto ao tipo de cuidado e apoio para necessidades da população em situação de rua. Diante dos resultados, como enfrentar o uso abusivo que alivia fome, solidão, sofrimento e depressão? Como cuidar do uso que gera fome, depressão, perdas afetivas, de saúde e autoestima? Uma história cíclica contada por pessoas em situação de extrema vulnerabilidade. Evidenciar a pluralidade simbólica do uso de drogas possibilita reflexões e novas formas de cuidar da população em situação de rua.

## Abstract (en)

Drugs have been consumed since ancient times, and their taboo has generated segregation and hindered the proper confrontation of harmful use. This study investigated the real and symbolic values of drug use for ill-housed people beyond pathologization, seeking to understand factors that hinder giving them up, even with intense losses and suffering. It also aimed to understand its implications for health care. This is a phenomenological qualitative research based on participant observation of ill-housed people care provided by the Street Clinic, interviews with ill-housed people and focus group with professionals from the Health Care Network in Rio de Janeiro city, Brazil, between March 2017 and July 2019. Results showed that “drug use” interacted with the research categories as follows: drugs are the main reason for living and staying on the streets; drugs motivate having financial activity; drugs cause abandonment of health treatment; drugs soothe and cause depression, suffering and hunger; drugs make them seek shelter and religion to “escape” using, but without adequate treatment hope leads to relapse and low self-esteem - which one want to get rid of, but few successes undermine hope. As expected, it is the reason for divergences among professionals regarding the type of care and support for ill-housed people. Given the results, how can we face abusive use that relieves hunger, loneliness, suffering and depression? How to take care of a use that generates hunger, depression, affective, health and self-esteem losses? A cyclical story told by people in situations of extreme vulnerability. Unveiling the symbolic plurality of drug use enables reflections and new ways of caring for ill-housed people.

## Abstract (es)

Desde la antigüedad las drogas se consumen y su tabú ha promovido la segregación y ha inhibido el enfrentamiento adecuado del uso nocivo. El objetivo fue descubrir valores reales y simbólicos del consumo de drogas por parte de la población sin hogar más allá de la patologización, buscando comprender factores que dificultan la renuncia, incluso con pérdidas y sufrimiento intensos. También tuvo como objetivo comprender sus implicaciones para el cuidado de salud. Esta es una investigación cualitativa fenomenológica, que implica la observación participante de la atención de la población sin hogar por el Consultorio en la Calle, entrevista de la población sin hogar y grupo focal con profesionales de la Red de Atención a la Salud, en el territorio del municipio de Río de Janeiro, Brasil, entre marzo/2017 y julio/2019. Los resultados mostraron que el “consumo de drogas” interactuó con las categorías de investigación de la siguiente manera: las drogas son la principal razón para vivir y permanecer en la calle; que motivan tener una actividad financiera; que hacen que las personas abandonen el tratamiento de salud; que aplacan y provocan depresión, sufrimiento y hambre; que hacen que las personas busquen refugio y religión para “escapar” de las drogas, pero sin el tratamiento adecuado, la esperanza se convierte en recaída y baja autoestima, de la que quieren deshacerse, pero los pocos éxitos socavan la esperanza. Correccionalmente, es motivo de desacuerdos entre profesionales en cuanto al tipo de cuidado y apoyo a las necesidades de la población sin hogar. Ante los resultados, ¿cómo podemos afrontar el uso abusivo que alivia el hambre, la soledad, el sufrimiento y la depresión? ¿Cómo cuidar el consumo que genera hambre, depresión, pérdidas afectivas, de salud y autoestima? Una historia cíclica contada por personas en situación de extrema vulnerabilidad. Resaltar la pluralidad simbólica del uso de drogas posibilita reflexiones y nuevas formas de cuidar de la población sin hogar.

## Introdução

Etimologicamente, droga significa folha seca, em referência aos medicamentos à base de vegetais. Na era moderna, “droga” perdeu essa conotação e remete a danos à saúde, violência, criminalidade, segurança pública e dependência química. Paradoxalmente, seu uso já foi associado a rituais religiosos, suportar adversidades, inibir a exaustão física e aumentar a carga laboral, tratar problemas de saúde e com finalidade recreativa. É certo que desde a antiguidade o homem consome drogas, no entanto, o tabu criado a respeito do assunto inibiu a circulação de informações na sociedade e, consequentemente, o enfrentamento relacionado ao uso nocivo ^1^.

Na atualidade, o uso de drogas provoca transtornos de saúde associados ao aumento no riscos de morbidade e mortalidade, podendo desencadear sofrimento substancial e prejuízos nas relações familiares, sociais e profissionais, que acomete 296 milhões de pessoas mundialmente, com tendência de crescimento ^2^, sendo responsável por 31 milhões de vidas saudáveis perdidas em 2019 ^2^, além dos homicídios atribuídos ao proibicionismo das drogas ^3^.

A última estimativa brasileira aponta 4,9 milhões de usuários entre 12-65 anos, sendo o álcool o mais preocupante, mostrando 2,3 milhões de pessoas com critérios para dependência ^4^. O *crack* foi associado a marcadores de exclusão social e considerado como grave problema de saúde pública ^4^ ^,^ ^5^.

A classe social não condiciona o consumo de drogas ^1^, mas a midiatização do *crack*, usado majoritariamente pela população em situação de rua ^5^, provocou inflexão na sociedade pelo imaginário “vicia na primeira tragada e mata em seis meses” ^1^ e contribuiu para construção social do usuário como doente mental desprovido de força de vontade e moralmente condenável, promovendo segregação e ódio ^6^.

A desigualdade social também marca o uso das drogas de diversas formas. Embora haja maior propensão de grupos socioeconômicos privilegiados iniciarem o uso, são os vulneráveis, pobres e excluídos que têm maiores chances de progredir do uso para transtornos relacionados à dependência e problemas de saúde mental ^2^, e serem considerados criminosos ao invés de usuários ^1^ ^,^ ^2^ ^,^ ^3^ ^,^ ^4^ ^,^ ^7^. Adicionalmente, a população em situação de rua é alvo de constantes experiências de desrespeito, indiferença e privação de direitos ^1^ ^,^ ^7^.

Vulnerabilidade socioeconômica e estigmas também implicam dificuldade no acesso às ações de promoção, prevenção e tratamento de drogas ^7^. De forma cíclica, quanto menor acesso, mais destrutivo e difícil de reversão são os efeitos decorrente do uso ^1^. Assim, exclusão social e uso de drogas formam um ciclo vicioso que se retroalimentam e afetam usuário e não usuário ^1^.

No Brasil, proibicionismo e repressão policial têm sido a forma prioritária de lidar com essa questão complexa ^3^ e, como consequência, morre-se mais por tiro na cabeça (código CID-10: S10) do que por overdose (código CID-10: X64), evidenciando necessidade de aprofundar questões relativas ao uso e à segurança pública.

Além dessas características, a população em situação de rua tem a particularidade da fome, privação de afeto, comorbidades não diagnosticadas ou tratadas com descontinuidade devido a baixa procura e dificuldade de acesso aos serviços ^7^ ^,^ ^8^, o que constitui desafios extras para o cuidado.

Este estudo objetivou descortinar valores reais e simbólicos do uso de drogas pela população em situação de rua para além da patologização, buscando entender fatores que dificultam sua renúncia, mesmo com perdas e sofrimentos intensos. Também visou compreender suas implicações para o cuidado em saúde.

Nesta pesquisa, valor simbólico relaciona-se com as múltiplas possibilidades de significação do mundo perceptivo-intuitivo ^9^, no qual uma coisa congrega uma pluralidade de sentidos que anteriormente estavam separados, mas são vivenciados como um todo. E o valor real é dado pelas representações construídas socialmente (droga como criminalidade ou fraqueza moral) ^1^ ^,^ ^6^ ^,^ ^10^, que muitas vezes se diferencia do mundo de sentidos vivenciados pela população em situação de rua.

As práticas tóxicas têm múltiplos significados na vida dos indivíduos: médico, religioso, místico, mágico, facilitador da comunicação e interação, e recreativo ^1^, todavia, não se nega seus efeitos destrutivos. Ampliar essas conexões pode contribuir com novas perspectivas de cuidado, ao passo que discuti-las sob um pano de fundo moralista prejudica tanto à compreensão quanto à adoção de políticas de controle mais efetivas.

## Método

Este artigo é oriundo de uma pesquisa, de perspectiva compreensiva e fenomenológica, sobre modo de vida e cuidado de saúde à população em situação de rua, no âmbito da Rede de Atenção à Saúde (RAS), em uma região programática do Município do Rio de Janeiro, Brasil, entre março de 2017 a julho de 2019.

Utilizou-se a combinação das seguintes técnicas de investigação: observação participante do cuidado prestado pelo Consultório na Rua (CnaR) por dois anos; entrevistas (E) com 24 pacientes do CnaR maiores de 18 anos e três grupos focais com profissionais de saúde da RAS que prestam atendimento à população em situação de rua no território.

O CnaR é a modalidade da atenção primária em saúde (APS) especificamente para cuidar da saúde da população em situação de rua, sendo complementado pela RAS ^8^.

A observação participante foi realizada no espaço de espera da população em situação de rua (para fins diversos); durante a prestação do cuidado de saúde no consultório do CnaR e território (cenas de uso de drogas, rua, outros equipamentos da saúde e assistência social) e reuniões de equipe do CnaR.

As entrevistas ocorreram após a observação participante, e a abordagem à população em situação de rua ocorreu durante a espera para atendimento no CnaR, sendo realizada em sala privativa. Não houve critérios prévios de seleção, objetivando a pluralidade de sujeitos. Exclusões foram por contraindicação clínica (tuberculose infectante ou atendimento de urgência). Partiu-se de roteiro prévio, mas conduzido de forma fluida, permitindo-se ser guiado pelos entrevistados e possibilitando o aparecimento do fenômeno na própria linguagem.

Participantes do grupo focal foram indicados pelos gerentes de cada equipamento, e todos os serviços que cuidam da população em situação de rua no território puderam participar, entretanto teve baixa participação daqueles vinculados à assistência social. Os encontros foram quinzenais, com duração de duas horas em local privativo, tendo participantes de serviços hospitalares, de urgência e emergência, da APS, do CnaR e de organização não-governamental de referência no atendimento ou proposição de atividades junto à população em situação de rua.

A análise orientada pelo método de narrativa fenomenológica, baseou-se nas anotações do diário de campo e transcrição dos áudios (entrevista e grupo focal). Adotou-se a abordagem etnográfica ^11^ de valorizar como a pessoa se narra, assim, as categorias foram estabelecidas a partir do que fazia sentido para elas, mesmo que depois fosse trabalhada e reinterpretada com auxílio de recursos teóricos ^1^.

Na perspectiva da população em situação de rua as categorias foram: “Droga e viver na rua”, “Droga e fome”, “Droga e renda”, “Rua das drogas”, “Droga como medicação”, “Droga como companhia”, “Droga e perdas”, “Fatores indutores do uso de drogas” e “Rua sem drogas”.

Quanto aos profissionais de saúde, a categoria “Droga e cuidado na perspectiva dos profissionais da RAS” mostra questões do cuidado relativo ao uso abusivo pela população em situação de rua, indicando fortes dissonâncias entre profissionais da RAS, ainda que não se resumisse a isso. A opção de destacar esse aspecto deve-se pelo desafio que ele representa ao cuidado em rede de pessoas excluídas socialmente e despossuídas de capital político, como a população em situação de rua, logo, desvela a complexidade desse cuidado.

Recorreu-se a utilização de fragmentos *ipsi literis* dos relatos dos participantes, assinalados entre aspas, para ajudar na compreensão da análise.

Esta pesquisa foi aprovada pelos Comitês de Ética em Pesquisa da Escola Nacional de Saúde Pública Sergio Arouca, Fundação Oswaldo Cruz (parecer nº 2.363.416), e da Secretaria Municipal de Saúde e Defesa Civil do Rio de Janeiro (parecer nº 2.403.259).

## Resultados

### Droga e viver na rua

O perfil dos entrevistados é apresentado no Quadro 1 e mostra uma população adulta diversa. O Quadro 2 contém excertos das narrativas da população em situação de rua e dos profissionais de saúde, segundo categorias de análise.

**Quadro 1:** Características sociodemográficas da população em situação de rua entrevistada. Rio de Janeiro, Brasil, 2019.

ENTREVISTA	IDADE (ANOS)	SEXO	ESCOLARIDADE	NATURALIDADE	TEMPO EM SITUAÇÃO DE RUA
E1	56	Masculino	Primário	Rio de Janeiro	Há 2 meses (a última vez)
E2	54	Masculino	Ensino Médio incompleto	Rio de Janeiro	Muitos anos
E3	45	Feminino	Primário	Espírito Santo	6 anos (a última vez)
E4	32	Feminino	Ensino Fundamental incompleto	Minas Gerais	Desde os 17 anos
E5	43	Feminino	Primário incompleto	Rio de Janeiro	“*Fumo crack há 15 anos. É mais ou menos o tempo que estou na rua*”
E6	20	Feminino	Ensino Fundamental incompleto	Rio de Janeiro	“*Vou pra casa, vou pra rua* (...) *Fico magrinha, vou pra casa. Fico 3 semanas em casa, volto pra rua*”
E7	30	Feminino	Ensino Superior incompleto	Minas Gerais	Há 18 anos
E8	47	Masculino	Ensino Superior incompleto	Espírito Santo	Há 10 anos
E9	35	Masculino	Primário incompleto	Rio de Janeiro	“*Usava cola desde 9, 10 anos de idade, por isso fui parar na rua, por um simples detalhe, uma primeira vez que se tornou a vida toda*”
E10	48	Masculino	Primário	Rio de Janeiro	5 anos (a última vez)
E11	53	Masculino	Ensino Médio completo	Rio de Janeiro	Há 20 anos
E12	35	Masculino	Iletrado	Rio de Janeiro	Há 2 meses. “*Pouco tempo*”
E13	35	Masculino	Ensino Fundamental completo	Rio de Janeiro	Há 2 anos. “*Foi depois da separação*”
E14	47	Masculino	Ensino Superior completo	Rio de Janeiro	Tem casa na comunidade. Fica na rua pra fumar *crack*
E15	45	Masculino	Ensino Fundamental incompleto	Rio de Janeiro	Desde 25 anos de idade. Mora algumas vezes na igreja (centro de tratamento). Na rua, dois meses dessa última vez
E16	29	Masculino	Ensino Médio completo	Rio de Janeiro	5 meses, dessa vez
E17	47	Masculino	Pós-graduação	Rio de Janeiro	Alguns anos. Já saiu da rua e já voltou
E18	66	Masculino	Ensino Médio completo	Rio de Janeiro	Quase 40 anos. “*Mas não ficou todo o tempo na rua*”
E19	42	Feminino	Ensino Médio completo	Rio de Janeiro	Poucos anos indo e vindo. Um mês direto
E20	35	Feminino	Ensino Fundamental completo	Rio de Janeiro	Vários episódios de rua - fica dias na rua
E21	56	Feminino	Ensino Fundamental incompleto	Rio de Janeiro	Mais de 30 anos
E22	57	Masculino	Primário incompleto	Minas Gerais	Mais de 30 anos. Está em abrigo agora por causa da cegueira
E23	37	Masculino	Iletrado	Rio de Janeiro	Desde os 10 anos de idade
E24	38	Feminino	Pós-graduação	Rio de Janeiro	Mais de 10 anos

**Quadro 2:** Relatos da população em situação de rua e de profissionais de saúde da Rede de Atenção à Saúde, segundo categorias de análise. Rio de Janeiro, Brasil, 2019.

CATEGORIAS	RELATOS DOS ENTREVISTADOS (POPULAÇÃO EM SITUAÇÃO DE RUA)
Droga e viver na rua	“*Comecei a usar droga aos onze anos e aos 29 anos comecei a usar crack. Quando usei crack, eu saí de casa e não voltei mais*” (E23) “*As outras drogas, eu não saia... continuei na minha casa. Tem como usar em casa, entendeu? Ligar para o Disk Droga ou ir e voltar*” (E19)
Droga e fome	“*Ai, eu vou ficar com fome de novo hoje.* [silêncio] *É muito ruim. Ainda mais que eu não sei ficar com fome. Aí começo a usar droga, droga, droga, droga, droga, droga; já vou sair daqui, ó, com o meu copinho* [para uso do crack] *ali dentro* [da sacola]*. Três dias e eu nem mexi nele. Eu vou sair daqui, vou raspar ele, vou ver se tem alguma coisa para sair dali, para já matar essa fome que eu estou*” (E7) “*Mas aí eu fui, com um sono danado, aí o que eu vou fazer? Quando acabar de garimpar aqui, eu vou comer, vou comprar um... dois pacote de biscoito, vou lá no fundo pra dormir. Caô, foi dois real mesmo! Peguei... Fumei* [crack]*. Fiquei ligadão. Trabalhei de novo pra fumar de novo*” (E15)
Droga e renda	“*Todo trabalho de catação é na intenção da droga*” (E1) “*Trabalho é pra arrumar dinheiro pra usar droga*” (E3) “*Eu prefiro garimpar em dois* [pessoas] *do que um, assim, você fuma mais rápido*” (E9) “*O problema tá em mim, não é o dinheiro*” (E4) “*Tô vendo ai... tenho planos de conseguir o meu BPC* [Benefício de Prestação Continuada] *para ter o meu canto e me afastar disso tudo aí* [drogas]” (E11)
Rua das drogas	“*É uma vida difícil* [drogadição]*, é uma vida árdua que não tem volta*” (E5) “*Eu sei que eu sou adicto, que é 24h por 24h. E vou ser adicto por toda a vida*” (E21) “*Se tiver assim uma experiência, ‘ah, vamos trocar o organismo dela todo para tirar o sangue’, eu ia querer na hora participar. Mas o meu maior problema na minha vida não é o HIV. O maior problema é o crack*” (E5) “*Acabou o crack tu já olha pro teu corpo e fica procurando alguma coisa de valor pra tu vender. Aí vem um e fala ‘não sou cracudão não, sou usuário’. Usuário nada, é cracudo, porque tu vendeu uma coisa, teu corpo, tira o chinelo do pé e dá para os outros para você fumar a pedra. Tu é cracudão*” (E10) “*Tu acha que* [o viciado] *acha maneirão ficar doidão, enchendo o saco da mulher, agredindo em casa? Não acha não. Só que o corpo dele já quer a cachaça. O negócio é a dependência do produto, senão teu corpo não vai ficar legal, tua mente não vai nada bem. É o cérebro, que está acostumado a receber aquele produto, aquela fumaça* [do crack] *desgraçada, maldita*” (E16) “*Se o crack está bom, eu vou atrás* (...) *não quero saber quem está vendendo, se é a ADA* [facção criminosa]*, ou essa Milícia; O crack está bom? ‘Por favor, me dá aí’. Até o padre, se tiver vendendo, eu falo ‘Me dá por favor?’*” (E2) “*O crack é uma droga absurda, você vê coisas que eu não desejo para um inimigo... demônios, é tipo morto-vivo, vê pessoas que amam você e entra naquela paranoia, cismando que elas querem te matar, entendeu?*” (E13) “*A droga não é problema, a rua é mais problema que a droga. Eu sei qual é a norma. Não pode estar em lugar de serviço usando a droga se não vou ser demitido, receber uma justa causa sem direito a nada*” (E1)
Droga como medicação	“*Eu fico assim* [com uso do crack]*, do jeito que eu estou, normal.* (...)*. E eu não consigo ter pensamentos ruins, sabe? Sem o crack complica* [enfatizou] *porque vou ter que aumentar muito as medicações legais, e aí vou virar um VEGETAL, cara. O crack consegue me estabilizar para eu ter uma vida quase normal. Só que a sociedade não entende que eu preciso* [enfatizou] *usar crack.* (...) *Aí, às vezes fumo crack, vou ler a bíblia, vou olhar alguns outros livros, eu sou viciado em livro, entendeu? Adorooo ler. E o único sofrimento que o crack provoca, além da recriminação e afastamento da família, é quando ele está ruim, porque gastou dinheiro à toa*” (E14)
Droga como companhia	“*Eu entrei numa depressão profunda, eu queria droga, droga, droga, que eu me sentia num refúgio. Mas quando acaba aquela sensação* [da droga]*, vem uma depressão que eu quero me matar, que eu quero me jogar, porque acabou o dinheiro, mas eu não roubo, eu não tenho onde pegar mais*” (E17) “*Como eu me decepcionei muito com o ser humano, a droga virou minha companhia*” (E24)
Droga e perdas	“*Matou a minha dignidade, a minha identidade, porque quando a gente está nas drogas, a gente perde o caráter, a gente perde a dignidade, a gente perde confiança, porque eu, hoje, eu ando arrumadinho, mas tinha dia que eu andava todo sujo, que nem um mendigo. As pessoas atravessavam, me viam, atravessavam para o outro lado, pensando que eu ia roubá-las*” (E11) “*Eu fico na abstinência, eu quero fumar crack, eu quero lá saber de se cuidar, de família, eu quero é isquerar, quero sentir o gosto do crack! Depois eu vou* [serviço de saúde]*, não vou... e ninguém faz eu vim. Hoje eu tinha tomado um banho... Foi diferente, por causa de que eu vou morrer e o crack vai continuar me matando. E o crack não vai deixar de existir, então eu tenho que vim e pensar em mim, porque se eu ficar só visando o crack, eu já tô seca, eu tô na pele e no osso*” (E6)
Fatores indutores do uso de drogas	“*...porque o consultório* [na Rua] *fica logo aqui na frente, do outro lado ficam uns 30* [usuários] *sentados na calçada, então venho com meu maço de cigarro no bolso e na vida de drogadição 'ei, dá um pedaço* [crack]*, eu te dou um pedaço e você me dá cinco cigarros'. Ou eu venho, minha mãe me dá o dinheiro da passagem, o dinheiro da passagem já é um escape pra vir pegar uma pedra, entendeu? Porque a boca de fumo é aqui na frente* [do Consultório na Rua]*, alguns passos que eu der, eu sei que eu tô na beira de ir ao uso novamente, entendeu? Se eu ver aquela fumaça saindo, ver alguém inalando, aquilo já mexe comigo. Então eu cortei caminho, vim pela rua de trás pra não ter... é aquele ditado: ‘o que os olhos não veem o coração não sente’. Então cortei o caminho pra não olhar aquilo ali e ter vontade de usar*” (E20)
Rua sem drogas	“*Cachaça dá muito sofrimento. E a forma é de querer sempre mais, né? Cachaça não tinha mais... Aí fui obrigada a largar, de abandonar!* (...) *Eu uso só o crack. Só esse, mais nada. E eu também tô saindo fora, como saí da cachaça, tô saindo fora também!*” (E21) “*Eu me senti, um dia, a pior coisa do mundo. Estava muito sujo, imundo, imundo, imundo. Fedendo! Olhei no espelho e falei: ‘-Meu Deus do céu, será que sou eu? Esse não sou eu.’ Aí eu falei: ‘Não, eu tenho que mudar de vida’. Aí fui, eu dormi uma semana na Cracolândia, esperando a abordagem, a abordagem veio... Da Guarda Municipal e da PM também, aí vim e falei: -‘Olha, eu quero mudar de vida. Me leve pro hotel* [social]*!’*” (E17)
CATEGORIA	RELATOS DOS PROFISSIONAIS DE SAÚDE DA REDE DE ATENÇÃO À SAÚDE
Droga e cuidado na perspectiva dos profissionais da RAS	“*Eu imploro pra ele pra ir pra clínica comigo, porque estão ali consumidos pela droga, e aí ele não quer ir. Eu falo assim: 'Ó, você vai perder o pé, vai dar bicho, vai ficar sem andar e aí é que não vai poder usar mais nada. Eu uso português claro, falando que vai morrer, pra ver se a pessoa se conscientiza que não é só droga que mata ele, mas aquele pé ali também pode matar*” (CnaR) “*A gente fica muito perdida lá na UPA... por causa da droga*” (UPA) “*A gente vê ‘a questão da droga’, mas às vezes é o que é menos danoso para aquela pessoa*” (CAPSad) “*Algo não muito elaborado porque o sujeito não dá conta. Ajusta o projeto as possibilidades da pessoa, mesmo que seja só reduzir os danos ou ouvir o que a pessoa tem pra falar ou ficar do lado enquanto ela chora. Se o projeto terapêutico inicial não der certo é importante recomeçar novamente e nunca desistir. Saber se vai dar certo, ninguém sabe, mas a gente tem que tentar*” (CnaR) “*Quando chega grávida* [nos serviços de saúde]*, ficam visando apenas a droga, sem olhar para aquela pessoa e investir em alternativas mais inclusiva para mãe e bebê. Já vai direto para o Conselho Tutelar!*” (CAPSad) “*A gente não consegue solucionar coisas que estão no âmbito da prefeitura, justamente porque ali é território um determinado partido, aqui é território de outro partido. E ficam pressionando porque precisam do leito. Se a gente conseguir encaminhar, convencer, conversar... ela vai voltar para a rua, que a gente sabe que é isso que acontece. É muito difícil se livrar da droga!*” (Hospital de Emergência) “*Tem os altos e baixos dos pacientes e os altos e baixos do profissional também... então, isso mexe comigo*” (CnaR)

CAPSad: Centro de Atenção Psicossocial Álcool e Outras Drogas; CnaR: Consultório na Rua; RAS: Rede de Atenção à Saúde; UPA: Unidade de Ponto Atendimento.

Fonte: elaboração própria.

Para 75% da população em situação de rua, o principal motivo para viver na rua foi o uso de drogas, sendo o *crack* a droga mais citada nesse contexto. Antes do *crack* era possível conviver com familiares usando outras drogas, mas a partir dele “*tem que sair de casa, porque ninguém quer conviver com cracudo*”. Uma vez na rua, é o *crack* que “segura” a pessoa nela, lugar do qual não consegue sair. Drogas como cocaína podem levar uma pessoa à rua, mas, geralmente, é o *crack* e o álcool, sobretudo a cachaça, pelo baixo preço praticado (Corote ou similar), que fixam a pessoa na rua. Por outro lado, o afastamento de casa diminui censuras e julgamentos do uso abusivo, bem como presenciar o sofrimento dos familiares (Quadro 2; E23 e E19).

### Droga e fome

Da totalidade, 12% declararam não passar fome na rua graças à ajuda de pessoas, estabelecimentos comerciais e religiosos, ou por estarem em locais com distribuição gratuita e itinerante de “quentinhas”.

Aqueles que não passam fome em situação de rua, não usam drogas ou usam parcimoniosamente, mantendo uma organização interna capaz de obter refeições gratuitas no território.

Em contrapartida, 88% dos que disseram passar fome, também recorrem às mesmas vias, mas alternam com uso intenso de drogas. Como um ciclo vicioso, a narrativa mais frequente foi a fome como uma força motriz para buscar algum dinheiro para se alimentar e, quando obtém o dinheiro, a fome cessa e a compulsão pela droga aumenta, a população em situação de rua dependente química acaba optando pelo uso de drogas com o dinheiro obtido. Depois que o efeito da droga passa, a fome volta e impulsiona atividades para obtenção de renda, cessando ao atingir valor suficiente para comprar mais droga. Por essa razão, muitos afirmam que “*droga alimenta a fome*”; “*usam mais droga pra fazer passar a fome*” ou “*escolhe o tipo de droga que não aumenta fome*” (Quadro 2; E7 e E15).

Portanto, população em situação de rua em uso compulsivo de drogas recorre, principalmente, à própria droga como estratégia de “alimentação”. Percebemos, inclusive, que a fome no contexto da população em situação de rua mostrou-se mais relacionada com o uso de drogas do que com o fato de viver na rua propriamente dito.

Sob outra perspectiva, o elevado custo das refeições em comparação com *crack*/cachaça de baixa qualidade, também impulsiona o ciclo entre droga e fome. Basta uma quantidade irrisória para adquirir a droga viciante: “*Na facilidade aí, é uma cachaça de 50 centavos, entendeu? É muito fácil comprar*”.

Apesar do ciclo que vivem, a fome é mostrada como muito ruim, e por isso precisam de ajuda, de outro modo comem do lixo ou vão para casa - para aqueles que mantêm laço familiar - para evitar o emagrecimento intenso. Aparência cadavérica é temida porque evidencia problemas de saúde e aumenta o afastamento da sociedade, prejudicando a ajuda voluntária de alimentos.

### Droga e renda

Uso abusivo de drogas mostrou-se também relacionado com formas de subsistência na rua: catação, ambulante, guardador de carro, Benefício de Prestação Continuada (BPC), aposentadorias e pensões, prostituição, tráfico e assaltos, pedir e “*manguear*”, segundo entrevistado, trata-se de contar história triste para obtenção de ajuda. A estreita relação entre uso abusivo e renda pode ser vista no Quadro 2 (E1, E3, E9), incluindo a síntese que identifica qual o problema e quem precisa de ajuda (E4).

A dificuldade de conciliar trabalho formal e uso excessivo foi o motivo pelo qual 12% dos entrevistados abandonaram o emprego antes de ir para rua. Contrariamente, a falta de renda contínua também se mostrou um obstáculo para replanejar a vida, sair da rua e se afastar do uso abusivo (E11).

### Rua das drogas

O uso de drogas foi um tema abordado por quase todos logo no início da entrevista, exceto um, que estava em abrigo, quase cego, que só falou sobre uso de drogas quando perguntado, mas disse que estaria usando se tivesse autonomia.

Todas as pessoas ouvidas já tinham usado ou estavam em uso, seja de *crack*, cocaína, cachaça, *thinner*, cola, *loló*, *ecstasy* ou um misto de várias drogas, denominado por eles como “*total flex*”. Dezessete por cento (17%) incluíram cigarro como droga, reconhecendo seu potencial viciante, mesmo que tratado distintamente pela sociedade. Maconha foi mencionada apenas como droga iniciante ou quando estavam domiciliados, pelo potencial de abrir o apetite, não era opção. A maioria dos autodenominados “*total flex*” revelou que depois do *crack* não têm mais desejo de outras drogas.

Dos usuários de *crack*, metade explicita fazer uso compulsivo, autodefinindo-se como adictos/viciados: “*Só uso droga direto, mais nada*”; “*Às vezes eu fico dois, três dias virada fumando crack*” (Quadro 2; E5, E21).

Outros definem o *crack* como o maior problema de suas vidas (Quadro 2; E5) e o termo “*cracudo*” é usado por quase totalidade dos entrevistados para evidenciar a visão pejorativa de si ou de terceiros sobre (si) usuários de *crack* (Quadro 2; E10).

Outras denominações do *crack* pelos usuários foram: “*maldito*”, “*horrível*”, “*destruição*”, “*vai me matar*”, “*acabou comigo*” e “*usar crack é pecado*”.

Outros vícios citados foram livros, jogos e mulheres. E um entrevistado resume dizendo que “*tudo é vício, tudo vira mau costume*”.

A partir das entrevistas e da observação participante pôde-se estabelecer um certo ranking das drogas, sendo *crack* e cachaça as mais usadas e mais perigosas, no sentido de levar à falta de domínio e ao descontrole e à dependência mais severa. A maioria que narra sobre dependência extrema, fissura, referem-se principalmente ao *crack* (Quadro 2; E16). Mesmo maldito, alguns disseram se importar bastante com a “qualidade” do *crack* (Quadro 2; E14).

A menção ao padre na fala do E2 (Quadro 2) não é displicente, marca vício e *crack* como antagônico à sacralidade, tal como a comparação acima de *crack* com pecado. Nenhuma outra droga foi relatada dessa forma. O *crack* afeta crenças e valores judaico-cristãos na população em situação de rua, acionando sentimento de culpa.

Cocaína, maconha, outras bebidas e cigarro são drogas tidas como mais leves, embora haja peculiaridades entre as pessoas. A principal diferença entre as “*leves*” e “*crack e cachaça*” (em estado de forte dependência) é que as últimas necessitam estar na rua para usá-las, principalmente *crack*.

Ele também é narrado como “*droga solitária*”, “*antissocial*”, porque a pessoa se isola para aumentar o efeito e não “*cortar o barato*”, e devido ao uso compulsivo e ininterrupto que provoca. Alguns descrevem a rotina na rua como “*droga, droga, droga*” e muitos sofrem com seu efeito negativo (Quadro 2; E13). Outras drogas fazem mais sentido “*em roda*” e é passível usar integrado à sociedade, se souber as regras (Quadro 2; E1).

Levando em consideração que o uso abusivo não é condição restrita da população em situação de rua, essa última fala destaca uma diferença importante entre pessoas domiciliadas e em situação de rua quanto ao uso de drogas: não “obedecer” às regras, fazendo uso em público e sem constrangimento.

O uso da *loló*, também “*droga de rua*”, foi presenciado durante as observações no território, mas só foi citada como preferencial por um entrevistado: “*uso muito loló, sem parar, e uso dorflex por causa das dores que dá*”.

### Droga como medicação

Depois de muito sofrimento narrado relacionado à droga, sobretudo ao *crack*, por quase a totalidade dos entrevistados, emerge um relato enfático sobre a importância do *crack* no controle dos pensamentos alucinógenos, das automutilações e da impotência sexual. Para ele, livro é seu vício e *crack*, sua medicação, e a única coisa ruim da droga é o julgamento da sociedade (Quadro 2; E14).

### Droga como companhia

De forma cíclica como a fome, foi frequente relatos sobre depressão e solidão e uso abusivo de drogas como forma de aplacar o sentimento de tristeza e desesperança, mas que passado o efeito, a depressão e solidão sobrevêm de forma mais intensa, necessitando recorrer novamente à droga para aliviar o sofrimento que será maior a cada “*onda*”. O sofrimento narrado é contundente (Quadro 2; E17 e E24).

O medo de ter a droga roubada pelo colega de rua contribui para o sentimento de solidão, mesmo que, aparentemente, esteja em grupo: “*não dá para confiar em ninguém, é só cochilar que eles fumam tua pedra*”, “*na rua não tem amigo não, te matam para roubar sua pedra*”.

O efeito tranquilizante e relaxante da droga é narrado de forma ambígua: o uso de droga ocorre para fugir da solidão, porém ela provoca afastamento e dificuldades de conviver com família e amigos; usa droga quando se aborrece, explode ou briga, mas passado efeito, e sem dinheiro para novas aquisições, vem a irritabilidade e o comportamento agressivo provocado pela abstinência: “*o dependente fica nervosão até usar droga. Ele precisa da droga*”.

Outros relativizam o uso: “*o problema não é droga, é ser pego*”, “*só usa quando quer usar*”, “*o crack é problema pra família, pelo preconceito*”, “*a sensação não dura nem cinco segundos, tem que curtir a onda que ela dá*”, “*é o corpo que quer, essa é a dependência do produto. Sem ele o corpo não fica bem, a mente não fica bem*”.

Resumidamente, a droga é vista de duas formas bem distintas, de maneira disfórica (predominantemente) ou eufórica (alguns poucos), e há ainda discursos que mesclam essas duas tendências, afirmando que ela tem efeito maléfico e benéfico interligados (ambivalência): ao mesmo tempo em que precisam e sentem benefícios ao usar, também reconhecem que são destruídos por ela.

### Droga e perdas

Segundo a população em situação de rua, os efeitos prejudiciais do uso abusivo são diversos e evocam sentimentos de perda em geral, podendo estar relacionados à saúde, ao bem-estar, à autoestima, ao dinheiro, ao emprego e à família, à residência e à dignidade, da seguinte maneira: (a) leva ao vício do jogo, faz jogar e perder dinheiro; (b) causa doenças, porque inibe ou prejudica o autocuidado, a alimentação e o uso de medicamentos regularmente; (c) faz emagrecer, porque a pessoa não se alimenta direito; (d) causa pânico, porque faz ouvir vozes e ter alucinações; (e) faz “perder” muitas coisas (dinheiro, família, emprego, bens); (f) faz perder a dignidade, porque leva ao crime (roubos na família, nas ruas) e porque transforma a autoimagem (fica sujo, dentes ruins, desleixado).

Outro constrangimento narrado é o pagamento de dívidas relacionadas às drogas por familiares, fazendo com que a pessoa decepcione aqueles que a amam.

O uso compulsivo pela população em situação de rua agudiza a perda de confiança sobre o controle de suas vidas e uma autoimagem despida de dignidade e autoestima, propiciando sentimento de inutilidade e sem local social próprio (Quadro 2; E6, E11).

Apesar do alívio que a droga proporciona, o preço elevado pelo sofrimento que ela gera, faz com que quase a totalidade deseje diminuir ou largar o uso, mas sentem-se incapazes pelas recaídas recorrentes, apesar dos tratamentos já realizados, e expressam esses momentos com ceticismo e tristeza: “*há muitas recaídas*”, “*é fácil recair*”, “*difícil resistir e de se livrar dela*”, “*impossível largar*”, “*dependência não tem cura*”, “*tombei de novo*”, “*me arrebentei de novo*”, “*tornei a cair*”, “*eu troquei o estudo pela droga e tropecei*”, “*larguei o crack, e derramei na cocaína*”.

### Fatores indutores do uso de drogas

Alguns entrevistados enunciaram que, além da situação de rua, certos lugares, certas companhias, ir à favela, a facilidade do “*disk drogas*” e a prostituição são fatores que aumentam o uso de drogas. A localização do próprio consultório do CnaR é vista com apreensão em momentos de abstinência pela proximidade de *cracolândias* (cenas de uso em espaços públicos) e pontos de venda (Quadro 2; E20). A prostituição é narrada como fator que “*enche o copo de crack*”.

No sentido oposto, o estado de preguiça é cobiçado porque, no entendimento de várias pessoas em situação de rua, sobretudo em dias chuvosos, é um incentivo e uma vitória abdicar de correr atrás de algum dinheiro para comprar a droga porque “*a preguiça venceu o vício*”.

### Rua sem drogas

Alguns poucos disseram ter se livrado das drogas, ou de algumas delas. Uma mulher expressou “*parei com todas as drogas, só uso o crack mesmo*”, enfatizando a conquista de ter deixado a dependência do álcool pelo sofrimento que sentia quando não tinha mais (Quadro 2; E21).

Outra mulher disse estar “*limpa*” do *crack* há anos mesmo vivendo na rua junto do companheiro usuário, e que conseguiu largar o vício após tratamento em centro de recuperação.

Um homem relatou estar sem usar *crack* há algumas semanas, depois de um plano inusitado de ir morar na *cracolândia* para conseguir vaga temporária no hotel da assistência social (Quadro 2; E17).

Dos que largaram ou desejam largar a droga, os motivos referidos foram: idade elevada, pois não é mais “*vistosa*” e não fica mais em grupinho; sofrimento que tinha quando não tinha a droga; pelo que vê de ruim no mundo das drogas; dar exemplo para filha usuária de droga; “*nada externo, é vontade própria, vem de dentro*”; “*sem regra ninguém consegue*”; “*pela dignidade que a abstinência traz*” (traduzido pelo asseio, uso de desodorante, cabelo cortado, barba feita e tratamento dentário). Alguns enfatizam terceiros como pretexto: “*mostrar que pode viver sem droga*”; “*surpreender as pessoas com abstinência*”; “*pensamento na filha*”.

As estratégias usadas para conseguir manter-se em abstinência foram: ocupar o tempo em espaço de convivência ou atividades culturais; “*dizer não, mesmo com ansiedade de usar*”; usar ansiolítico e “*rezar muito para curar o vício*”; “*ter fé, mas tem hora que não aguenta, foge e usa droga novamente*”, “*igreja*”, além de buscar apoio para sair da rua (com familiar, abrigo, comunidade terapêutica, hotel). Uma dificuldade frequentemente narrada foi que “*abstinência dá fome*”.

Acerca de abrigos e comunidades terapêuticas (equipamento de cunho evangélico, voltado para pessoas com problemas de uso) verificam-se ambivalência em relação ao apoio para tratamento do uso abusivo de drogas. Abrigos facilitam o afastamento do ambiente viciante, porém há omissão no controle ou até maior acesso em alguns locais: “*só tem drogado lá e só se fala sobre drogas entre pacientes*”.

Para outros, tratamento em comunidade terapêutica não funciona porque “*não pode falar sobre drogas. Meu problema é droga, como não posso falar?!*”; “*Só não usa enquanto estiver lá, quando sair, volta pra todos contatos e a droga. Não dá para viver a vida toda em comunidade terapêutica!*”.

### Droga e cuidado na perspectiva dos profissionais da RAS

Por parte dos profissionais, houve divergências de crenças e valores sobre uso abusivo pela população em situação de rua, refletindo em discriminação e formas de cuidado (Quadro 2; CnaR, UPA, CAPSad, Emergência). Alguns mostraram preferência por tratamentos mais repressivos e restritivos de autonomia, enquanto outros advogavam pela abordagem de redução de danos, por prover “*o cuidado com o que é possível*”, repactuação dos votos de confiança e reconstrução de vida. Também foi constatado insegurança e desespero de profissionais diante de certos momentos de drogadição intensa, usando “*ameaça*” como estratégia de desespero (Quadro 2; CnaR). Outros tentam, pela amizade e camaradagem, seduzir a população em situação de rua a interromper o uso para dar continuidade do cuidado, seja no comparecimento para exames marcados ou curativo.

A propósito, alguns advertem que quando a pessoa em situação de rua chega para atendimento nos equipamentos o foco é restrito a droga, mesmo que não seja uma demanda dela (Quadro 2; CAPSad).

Cuidado em relação ao uso abusivo mostrou ser um grande desafio para diferentes dispositivos, sobretudo entre áreas da saúde e assistência social, implicando em desconhecimento e descontinuidade de estratégias e desafios para ações intersetoriais.

O sentimento de desolação diante episódios de uso contínuo e sofrimento do *crack* também foi vivenciado por alguns pesquisadores durante atividade de campo.

Gestante em uso de drogas e situação de rua foi o tema de maior tensionamento entre dispositivos do território, tendo direções opostas nas estratégias de cuidado: favorável ao uso do conselho tutelar para retirar a criança da mãe assim que nascesse e crítica do uso do conselho como poder de polícia e punitivo (Quadro 2; CAPSad).

Outro ponto dissonante entre profissionais foi relacionado ao apoio para obtenção do BPC pela população em situação de rua e fornecimento de refeição pelos equipamentos da RAS, por conta do uso abusivo de drogas. Alguns acreditam que esses apoios são potencializadores para uso abusivo, pois a pessoa em situação de rua não precisaria verter parte do dinheiro que obtém para comprar comida ou mesmo se organizar para obtê-la. Para outros, comida e BPC são parte integrante do cuidado de alguns. Alegam que as poucas refeições disponíveis não iriam alterar a rotina da população em situação de rua e o BPC auxiliaria na saída da rua e reconstrução da vida.

Recaídas e uso abusivo, e suas implicações na descontinuidade e piora da saúde, geram sentimentos distintos entre profissionais. Uns olham com mais naturalidade os “*altos e baixos*”, “*porque a vida é assim: hoje ela estava chorando e no dia seguinte aparece toda serelepe*”. Contudo, outros compartilham os mesmos sentimentos de “*esperança e desesperança*” vividos pela população em situação de rua.

A complexidade do projeto terapêutico pode ser percebida pela dissonância entre equipe e questionamentos sobre até que ponto as pessoas conseguem ir ou se a equipe não está “*ficando muito acostumada e conformada*” em relação às recaídas.

Foi unanimidade por parte dos profissionais, o reconhecimento de que dependente químico em situação de rua sofre dupla carga de preconceito e discriminação na relação com os serviços da RAS. Igualmente unânime, foi o impacto que o crescimento do neoliberalismo teve na precarização e descontinuidade de políticas públicas, de oferta de serviços e das contratações e condições de trabalho dos profissionais, culminando no aumento de práticas contraditórias relacionadas às drogas, entre serviços da RAS.

## Discussão

Este artigo objetivou estudar os valores real e simbólico do uso de drogas pela população em situação de rua para ampliar a compreensão e refletir sobre cuidado em saúde de quem vive sob efeitos devastadores da exclusão e degradação social: além do estigma “*morador de rua*”, soma-se o de “*drogado, criminoso*” ^1^ ^,^ ^3^ ^,^ ^6^.

Os efeitos destrutivos do uso abusivo são mais difíceis de reverter quando maior a precariedade de acesso a bens econômicos e simbólicos, incluindo relações que proporcionam sustentação afetiva e moral ^1^ ^,^ ^2^. Se é a forte dependência que liga as pessoas a rua (rua de drogas, drogas e viver na rua), é essencial o acesso às políticas de proteção social (comida, renda, trabalho etc.) e cuidado baseado em relações de vínculo e afeto, para reverter o aumento vertiginoso de pessoas em situação de rua ^2^ ^,^ ^3^. Contudo, o cuidado dos usuários é atravessado por dificuldades de acesso, privação de direitos básicos ^4^ ^,^ ^7^ ^,^ ^8^ ^,^ ^10^ e discriminação ^7^.

Quando não se tem nada, a droga é tudo. É uma tentativa desesperada de escapar de um cotidiano insuportável, mesmo que o uso contínuo (“*droga, droga, droga*”) só aumente o desprezo social e a degradação moral.

A partir das categorias, percebeu-se que, frequentemente, elas aparecem em oposição (droga que alimenta e dá fome; droga que alivia a tristeza e aumenta a depressão; etc.), todavia, sem contradição. A fenomenologia possibilita compreender a realidade humana com múltiplas possibilidades de significação ^9^ e compreender o valor simbólico do uso abusivo para cada sujeito aumenta o autoconhecimento e auxilia na construção de projetos terapêuticos singulares adequados, implicando em menores recaídas e descrédito.

Os resultados mostram que população em situação de rua que faz uso abusivo (“*só droga direto*”) sofre mais fome, solidão, depressão, dificuldade de manter trabalho e renda para bens essenciais e sofrimento pelo sentimento de derrota. Não ter acesso às necessidades fundamentais condiciona sentimento de não-pertencimento, não-reconhecimento e falta de dignidade social ^1^, necessitando cuidado para além das drogas (profissional CAPSad) e, consequentemente, para além da redução de danos. É fundamental políticas públicas protetivas e articulação intersetorial para acesso aos direitos sociais constitucionais (comida, trabalho e renda) ^1^ ^,^ ^4^ ^,^ ^7^ ^,^ ^8^ ^,^ ^10^.

A prisão do vício não se limita ao abuso da substância, mas amplifica o abandono afetivo e social sofrido, e a privação de afeto aumenta a busca pela satisfação imediata proporcionada pela droga - sobretudo *crack* - para fugir da realidade intragável e da incapacidade de formular e planejar futuros ^1^.

Acerca das categorias fome/trabalho/renda (pela população em situação de rua) e fornecimento de comida/auxílio para obtenção do BPC (por profissionais de saúde), Souza ^1^ destaca que ter renda/trabalho é condição fundamental na modernidade para ter reconhecimento social e dignidade. Brito & Silva ^7^ mostraram a desvalorização da própria população em situação de rua contra sua forma de trabalho/renda usual (catação; prostituição), em contraponto com trabalho formal, desvelando o trabalho institucionalizado como símbolo de reconhecimento social e autoestima. A compreensão do fornecimento de alimentação/auxílio para BPC por alguns profissionais, alicerçada na crença de que se tiver dinheiro/comida comprará droga ao invés de comida (valor real), desvela uma prática moralista, calcada na midiatização e culpabilização moral do usuário ^6^ e de privação/violação de direitos sociais, humanitários e de cidadania.

Fome é necessidade básica de todo ser vivo e, apesar de parecer ser assunto pouco controverso, o uso de drogas afeta a credibilidade da população em situação de rua, realimentando o ciclo punitivo e restritivo de acesso aos direitos e bens básicos. Ter acesso ao BPC, que lhe é direito, pode ser condição primordial para reconstrução da dignidade e vislumbrar um futuro de esperança.

Pesquisa prévia com população em situação de rua e usuários de *crack* ^1^ também observou que profissionais boicotavam acesso aos benefícios pela possibilidade da remuneração financiar a compra de drogas e manutenção do vício, evidenciando maior preocupação com a descontinuidade do uso do *crack* do que a reinserção social proporcionada pela renda ^1^. Para Souza ^1^, reinserção social e, portanto, acesso aos direitos sociais, é condição fundamental para sair do ciclo vicioso, possibilitado pela reconstrução de um novo horizonte temporal, que possibilite sair do “*aqui e agora*” da “*droga, droga, droga*”.

Todavia, instituições de tratamento para usuários tendem a robustecer os preconceitos, adotando medidas coercitivas e discriminatórias, em detrimento de direitos de cidadania ^10^. Segundo Medeiros ^10^, elas deveriam combinar saberes interdisciplinares e analisar as particularidades e significados simbólicos do uso de drogas e do contexto sociocultural dos usuários.

“*Mulher*”, “*situação de rua*” e “*uso abusivo*” personifica triplo preconceito, fruto do conservadorismo e sexismo que persiste na sociedade ^7^, ao invés de serem tratadas como sujeitos que vivem com as desvantagens das assimetrias sociais de gênero ^1^. Mulher grávida ou que se prostitui representa antítese do imaginário social (valor real): amor incondicional por filhos e marido, decepcionando expectativas vinculadas aos papéis sociais de mãe e esposa ^1^, como consequência, são tratadas com julgamento e rigor da mão pesada do poder público. Grávida usuária e em situação de rua vive a interseccionalidade de ser prostituta e incapaz de assumir a responsabilidade da criança. O conselho tutelar é contactado para tirar a criança da mãe tão logo ela nasça, sem qualquer perspectiva de apostar naquele vínculo e novos futuros (profissional CAPSad).

A reflexão sobre o que é “*vicio*” (jogos/mulher/livro) e o que é lícito/ilícito (“*cigarro*”; “*droga como medicamento*”) feita pela população em situação de rua, evidência que ela não é alienada e que o conservadorismo restringe o debate científico e a capacidade de implementar soluções adequadas, produzindo mais preconceito e assimetria da atuação da segurança pública sobre usuários ^1^ ^,^ ^2^ ^,^ ^3^ ^,^ ^4^ ^,^ ^5^ ^,^ ^6^. Menções religiosas “*droga como pecado*” ou similares (Rua das drogas) exprimem a competição entre saúde e práticas religiosas, podendo prejudicar o cuidado dos usuários ^7^.

A capilarização do neoliberalismo no Brasil provocou pioras nas condições de vida e trabalho, seja pela privatização para contratação e gestão dos serviços de saúde pelas organizações sociais, seja pelas medidas restritivas de investimento em saúde (teto dos gastos/arcabouço fiscal), promovendo impactos devastadores na proteção social e sustentabilidade dos sistemas públicos e, por conseguinte, nos cuidados em saúde ^12^. Aliado à persistente desarticulação entre equipamentos da saúde e da assistência social que integram a RAS, intensificaram dissonâncias entre profissionais que cuidam da população em situação de rua ^12^ ^,^ ^13^ e sentimento de não saber lidar com desafios do cuidado. Recentemente, tivemos retração do avanço neoliberal sobre políticas de proteção social e trabalhistas, mas ameaças sempre persistem.

Evidenciou-se também um campo de disputa do paradigma clínico: equipamentos da APS tendem para redução de danos, enquanto hospitais gerais, instituições de abrigamento e Comunidades Terapêuticas são mais voltados para abstinência ^13^, levando rupturas nos projetos terapêuticos, reforçando sentimento de fracasso pelas sucessivas recaídas, tanto pela população em situação de rua quanto pelos profissionais. O fracasso recai sobre o sujeito, sem perceber que são fruto de políticas e financiamentos desconexos ^1^ e do afastamento do serviço com aquele que sofre ^13^.

Consequentemente, vários profissionais não sabem o que é melhor para o paciente quando envolve toxicomania, gerando angústia na equipe, principalmente quando a vida está em jogo ^13^.

O ciclo da droga e os associados a ela - fome, perdas, solidão e sofrimento -, mostrado nos resultados desta pesquisa e outros ^1^ ^,^ ^4^ em consonância, somado a informação de que 80% dos entrevistados em pesquisa nacional desejavam receber tratamento para o uso abusivo de drogas ^5^, impõe reflexões sobre formas de cuidar e integração de abordagens entre serviços.

A abordagem de redução de danos, que visa reduzir ou prevenir danos causados pelo uso sem necessariamente passar pela abstinência, tem suas restrições diante da grande necessidade da população em situação de rua. De forma prática, como pensar em redução de uso ou substituição do *crack* por outras substâncias, como a maconha, sem incluir a garantia de refeições?

Contudo, existe a fome e a fome de sentido de vida. Distintas formas de lidar com as coisas ^9^, demandam que profissionais conheçam as singularidades dos usuários para refletir, com eles, estratégias possíveis e essenciais para suas vidas ^10^ e de sentido de existência ^6^ ^,^ ^9^.

A falta de recursos necessários para responder às demandas da população, amplificada com precarização e desvalorização do trabalho, também afeta profissionais ^12^, provocando desesperança e impotência, os “baixos” na gangorra da vida.

Quanto às limitações do estudo, o maior uso do *crack* pelos entrevistados pode ser reflexo das características do território da pesquisa, com *cracolândias* e boa qualidade da droga reportada. A longevidade da observação participante facilitou a transferência da confiança da população em situação de rua pela equipe do CnaR para os pesquisadores, possibilitando aprofundamento de temas dolorosos e sabidamente discriminatórios pela população em situação de rua.

Por outro lado, os grupos focais deram-se em período de crises políticas e econômicas que contribuíram para aumentar ameaças de emprego, descontinuidade de políticas progressistas e desfinanciamento dos serviços públicos, impactando a confiança dos trabalhadores e silenciando certas tensões vividas.

## Conclusão

Nesta pesquisa, a categoria “*droga*” teve forte interação com “*motivo de ir e manter-se na rua*”, “*fome*”, “*trabalho e renda*”, “*abrigo*”, “*abandono de tratamento*”, “*cuidado de saúde*”, “*saúde mental*”, “*sofrimento*”, “*preconceito*”, “*percepção de si*” e “*religiosidade*”, tendo sido a de maior interação entre as categorias de toda pesquisa. Pela limitação do artigo alguns tópicos foram resumidos.

Essa interação acontece da seguinte forma: para muitos, a droga é o principal motivo pelo qual a pessoa vai e mantém-se na rua; que motiva atividade financeira; que faz abandonar tratamento de saúde, incluindo medicamentoso; que aplaca e provoca depressão, sofrimento e fome; que faz buscar abrigo e religião para “fugir” das drogas, mas sem tratamento adequado, a esperança transverte-se em recaída; que transforma de forma pejorativa a autoestima e visão de si, da qual se deseja livrar, mas os poucos êxitos minam a esperança. De forma correlacional, a droga também é o principal motivo das reticências e dualidade dos profissionais de saúde tanto em relação ao fornecimento das poucas refeições disponíveis e do auxílio no acesso ao BPC e ambivalências quanto ao tipo de cuidado prestado no que concerne a ela.

Diante desses resultados, como enfrentar o uso abusivo de droga que mata fome, solidão, sofrimento, pensamentos alucinógenos e depressão? Como cuidar do uso de drogas que gera fome, depressão, sofrimentos, perdas afetivas, laborais, financeiras, de saúde e autoestima? Uma história cíclica contada por pessoas em situação de extrema vulnerabilidade.

Evidenciar a pluralidade das questões que envolvem uso abusivo de drogas possibilitará aprendizados mútuos e novas reflexões sobre o projeto terapêutico da população em situação de rua. Nunes & Brito ^12^ salientam para o potencial de transformação a partir das ambiguidades e contradições.

Quebrar ciclos viciosos requer abordagens integradas e abrangentes e, acima de tudo, acabar com a exclusão causada pela marginalização, discriminação e estigma relacionada às drogas ^1^ ^,^ ^2^ ^,^ ^3^ ^,^ ^4^ ^,^ ^5^ ^,^ ^6^ ^,^ ^7^, ampliando círculo de cuidado e compaixão ^7^ ^,^ ^12^.
